# Emerging role of VCP/p97 in cardiovascular diseases: novel insights and therapeutic opportunities

**DOI:** 10.1042/BST20200981

**Published:** 2021-01-13

**Authors:** Hongyang Shu, Yizhong Peng, Weijian Hang, Ning Zhou, Dao Wen Wang

**Affiliations:** 1 Division of Cardiology, Department of Internal Medicine, Tongji Hospital, Tongji Medical College, Huazhong University of Science and Technology, Wuhan 430000, China; 2 Department of Orthopedics, Union Hospital, Tongji Medical College, Huazhong University of Science and Technology, Wuhan 430000, China

**Keywords:** cardiovascular diseases, iNOS, mitochondria, VCP/p97

## Abstract

Valosin-containing protein (VCP/p97) is a member of the conserved type II AAA+ (ATPases associated with diverse cellular activities) family of proteins with multiple biological functions, especially in protein homeostasis. Mutations in *VCP/p97* are reportedly related to unique autosomal dominant diseases, which may worsen cardiac function. Although the structure of VCP/p97 has been clearly characterized, with reports of high abundance in the heart, research focusing on the molecular mechanisms underpinning the roles of VCP/p97 in the cardiovascular system has been recently undertaken over the past decades. Recent studies have shown that VCP/p97 deficiency affects myocardial fibers and induces heart failure, while overexpression of VCP/p97 eliminates ischemia/reperfusion injury and relieves pathological cardiac hypertrophy caused by cardiac pressure overload, which is related to changes in the mitochondria and calcium overload. However, certain studies have drawn opposing conclusions, including the mitigation of ischemia/reperfusion injury via inhibition of VCP/p97 ATPase activity. Nevertheless, these emerging studies shed light on the role of VCP/p97 and its therapeutic potential in cardiovascular diseases. In other words, VCP/p97 may be involved in the development of cardiovascular disease, and is anticipated to be a new therapeutic target. This review summarizes current findings regarding VCP/p97 in the cardiovascular system for the first time, and discusses the role of VCP/p97 in cardiovascular disease.

## Introduction

Valosin-containing protein (VCP/p97) was firstly identified in 1982 by Moir et al. [1], when they genetically screened *Saccharomyces cerevisiae*. This protein is strongly associated with cell cycle arrest and was therefore called Cdc48 (cell division cycle 48). In *Drosophila* [2], VCP/p97 is localized to the endoplasmic reticulum (ER) and belongs to the type II AAA+ ATPase family; hence, it is also referred to as the transitional endoplasmic reticulum ATPase (TER ATPase). Mammalian homologs have been reported as a precursor of the small peptide valosin [3], known as valosin-containing protein (VCP/p97). The term VCP/p97 has been used to represent this protein in this review.

VCP/p97 is a molecular chaperone involved in the endoplasmic reticulum-associated degradation pathway (ERAD) [4]. More specifically, VCP/p97 is recruited to the ER membrane by binding with membrane adapters, including UBX domain-containing protein 1 (UBXD1), p47, and nuclear protein localization protein 4 (Npl4) [5–7]. VCP/p97 captures and extracts misfolded proteins through its putative channel by ATP hydrolysis, and subsequently targets misfolded proteins for proteasomal degradation. In addition to ERAD, VCP/p97 is involved in most organelle-mediated protein degradation processes, including the degradation of proteins in the outer mitochondrial membrane [8], nucleus [9] and co-translational degradation in ribosomes [10]. Additionally, VCP/p97 also plays a critical role in DNA damage [11]. In summary, VCP/p97 is a multifunctional protein that has considerable impact on protein metabolism and intracellular homeostasis.

Among various tissues, VCP/p97 is present in cardiac tissues in high abundance, only second to skeletal muscle. Dilated cardiomyopathy is observed in patients with IBMPFD (inclusion body myopathy (IBM), Paget disease (PDB), neurological components frontotemporal dementia (FTD)) who experience pain due to *VCP/p97* mutations [12]. Silencing of TER94 (a homolog of VCP/p97 in *Drosophila*) also seriously damages heart function in *Drosophila* [13], which together highlights the importance of VCP/p97 in the heart. Current research has initially revealed the role of VCP/p97 in ischemia–reperfusion and hypertensive heart disease, but there is still controversy. This review summarizes the role of VCP/p97 in the cardiovascular system, particularly focusing on the underlying pathophysiological mechanisms, and sheds light on the remaining problems involving basic research surrounding cardiovascular diseases, thus aiming to raise awareness and interest concerning VCP/p97 in the cardiovascular system.

## The *VCP/p97* gene

The *VCP/p97* gene is located on chromosome 9p13.3, and contains 17 exons. More than 50 missense mutations involving the *VCP/p97* gene are related to unique autosomal dominant diseases [14]. These diseases are collectively referred to as ‘multisystem protein diseases', including IBMPFD [15], which denotes a combination of inclusion body myopathy (IBM), Paget disease (PDB), and neurological components frontotemporal dementia (FTD). These patients eventually die from muscle weakness and heart failure.

Single missense mutations of *VCP/p97* can lead to IBMPFD [16]. These pathogenic single missense mutations are mainly located at the N-terminus of *VCP/p97* (N domain or D1 domain). Among the mutations, the R155H substitution is the most common mutation in IBMPFD patients [17], while the A232E mutation results in the most serious clinical manifestations. *VCP/p97* mutations also account for 1%–2% of the familial amyotrophic lateral sclerosis (ALS) cases [18]. Pathogenic *VCP/p97* mutations lead to reductions in SUMOylation and the formation of VCP/p97 hexamers under stress. They also affect cofactor binding and degradation of endoplasmic reticulum-associated proteins, which ultimately renders cells vulnerable to stress [19]. Additionally, adenine nucleotide transferase is dysregulated due to pathogenic mutations involving *VCP/p97*, resulting in reduced ATP levels [20]. Utilization of the Cre-LoxP technology for excision of the *VCP/p97* R155H missense mutation was anticipated as a correctional approach for this mutation [21], but currently there are no effective treatments for these genetic mutations.

## The VCP/p97 protein

The VCP/p97 protein has 806 amino acids, and a predicted molecular weight of 97 kDa [22].

It is markedly abundant, accounting for ∼1% of the total cellular proteins. Generally, VCP/p97 is soluble in the cytosol, and is found to be associated with membrane structures, including the endoplasmic reticulum [23], golgi apparatus [24], mitochondria [25], and endosomes [26]. Additionally, VCP/p97 has been shown to play a role in the nucleus, mainly related to nuclear protein quality control [27].

VCP/p97 belongs to the type II AAA+ ATPase family [28]. It usually forms hexamers in cells, and its assembly is independent of ATP [29]. VCP/p97 has two ATPase domains, referred to as D1 and D2, which are connected by a short peptide linker [30]. The D1 domain is also connected by the N-D1 linker to the N-terminal domain, which mediates interactions between VCP/p97 and most of its cofactors, including Ufd1-Npl4, p37, p47, and so on [31–33]. Additionally, there are certain other proteins that bind to the C-terminal tail linked to the D2 domain. For example, UBXD1 and phospholipase A2-activating proteins occupy the C-terminus of VCP/p97 [25,34].

To date, at least 40 proteins that interact with VCP/p97 have been identified [35,36]. These proteins usually have a common binding motif and conserved binding modules, such as UBX (ubiquitin regulatory X) domain, UBXL (UBX like) domain, VIM (VCP/p97-interacting motif), and VBM (VCP/p97-binding motif) [37]. For example, the cofactor alveolar soft part sarcoma locus (ASPL), which has the highest affinity with VCP/p97, relys on its extended UBX domain (eUBX) [38]. These bind to different sites on VCP/p97, and then guide VCP/p97 to different membrane structures in order to perform a variety of biological functions. The VCP/p97 hexamer has many exposed domains, thereby allowing combinations of multiple cofactors at the same time; hence, the functions of VCP/p97 are diversified, and the regulation of VCP/p97 can be quite precise.

## VCP/p97 and cardiovascular diseases

Imbalance in protein homeostasis is a hallmark of various cardiovascular diseases [39], including myocardial infarction, heart failure, and diabetic cardiomyopathy. Considering the core role of VCP/p97 in protein homeostasis, VCP/p97 is thought to be involved in the cardiovascular system. For example, *VCP/p97* K524A transgenic mice exhibit cardiomyopathy with the accumulation of ubiquitinated proteins [10]. In addition to protein homeostasis, VCP/p97 also plays a vital role in the maintenance of mitochondrial function and the promotion of cardiomyocyte survival [40]. In the following sections, we have summarized certain basic findings concerning VCP/p97 in cardiovascular diseases.

## VCP/p97 and ischemia/reperfusion injury

### VCP/p97 and iNOS

**Figure 1.**
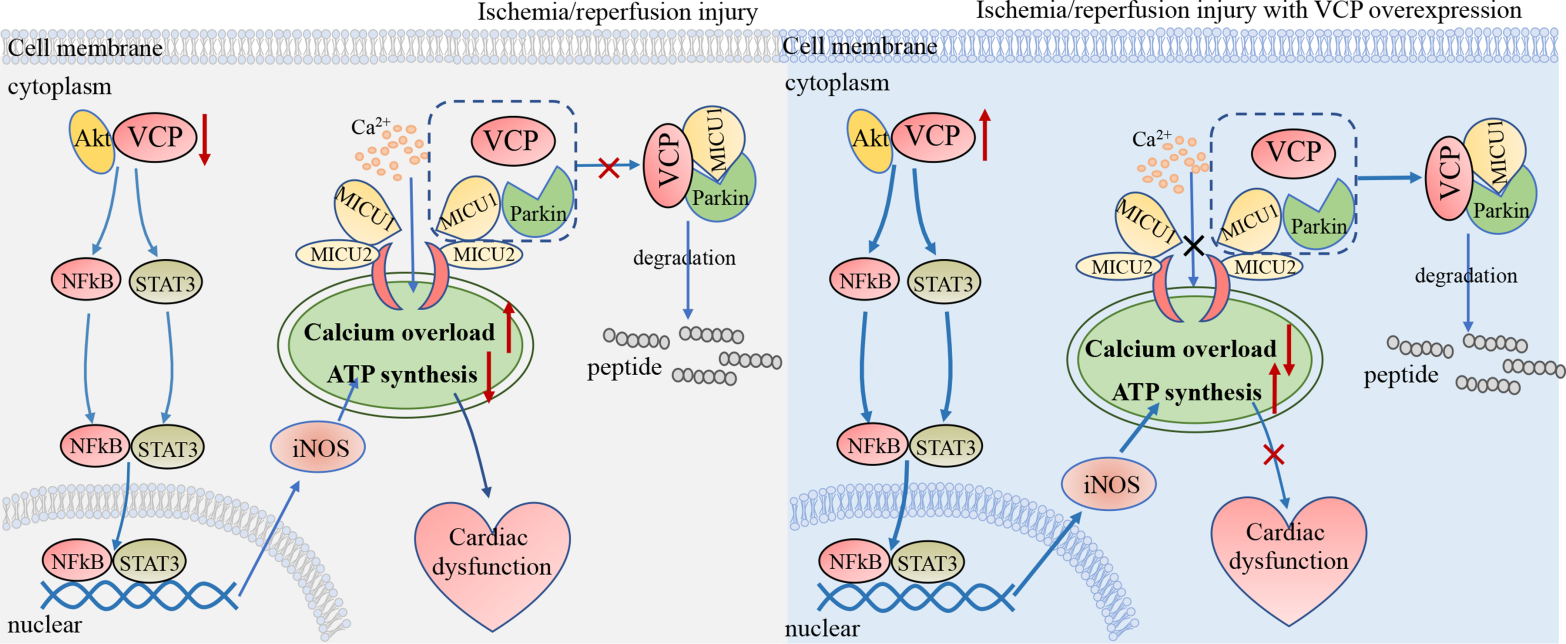
VCP's effects on cardiac ischemia/reperfusion injury. The decreased VCP in ischemia reperfusion leads to a decrease in the physically interaction of VCP with Akt and the activation of NF-kB and STAT3 in cardiomyocytes, NF-kB and STAT3 are the transcriptional factors of iNOS, which has been proven to improve the respiratory function of mitochondria. In addition, the physically association of VCP, MICU1, and Parkin decreased, which reduced the degradation of MICU1, resulting in the continuous opening of mitochondrial permeability transition pore, calcium overload and decreased respiratory function in the mitochondria. Therefore, the decline of iNOS and the respiratory function of mitochondria jointly promote the cardiac dysfunction under ischemia–reperfusion. The overexpression of VCP enhances the interaction of VCP and Akt, which results in an increase in NF-kB and STAT3 into the nuclear, and the production of iNOS, thereby enhancing the mitochondrial respiratory function of cardiomyocytes. Meanwhile, the physically interaction of VCP, MICU1 and Parkin is enhanced under conditions of VCP/p97 overexpression, which accelerates the degradation of MICU1, reduces the opening frequency of mitochondrial permeability transition pore, and inhibits mitochondrial calcium overload. Thus the increase in iNOS production and the improvement of mitochondrial respiratory function together protect the cardiac dysfunction under ischemia–reperfusion.

Ischemia–reperfusion is a process in which part of the myocardium receives insufficient blood supply due to clogged blood vessels in the ischemic phase, and then blood returns to the ischemic area in the reperfusion phase [41]. Both ischemia and reperfusion cause myocardial tissue damage [42,43]. Ischemic preconditioning for ischemic myocardium effectively relieves ischemia/reperfusion injury [44]. In the ischemic preconditioning process, endogenous NO produced within the myocardium regulates myocardial contraction and vessel vasodilation, thus reducing the area of myocardial infarction and improving endothelial function [45]. The synthesis of NO is mainly mediated by inducible NO synthase (iNOS) [46], which places iNOS in a crucial position in ischemic preconditioning [47]. VCP/p97 is a protective factor in ischemia/reperfusion injury, supported by a 50% reduction in the myocardial infarction area in VCP/p97 transgenic mice compared with that in wild-type mice after ischemia/reperfusion injury [48]. Moreover, overexpression of VCP/p97 in cardiomyocytes significantly reduces celandine-induced myocardial apoptosis [49]. Further mechanistic studies have revealed that VCP/p97 exerts its effects encompassing myocardial protection by elevating the production of NO via iNOS (Figure 1). VCP/p97 promotes iNOS expression in a dose-dependent manner, which largely relies on NF-kB (Figure 1), as the proteolytic effects of VCP overexpression are abolished by the addition of the NF-kB inhibitor, SN50 [49].

### VCP/p97 and mitochondria

Heart is a high-energy consumption organ, which relies on abundant mitochondria to provide energy; more than 90% of the ATP requirement is fulfilled by mitochondrial aerobic oxidation [50]. Patient-derived VCP/p97-mutant fibroblasts display low mitochondrial membrane potential and reduced ATP levels [51]. Mutation, or lack of VCP/p97 in neurons, aggravates mitochondrial dysfunction, resulting in reduced ATP synthesis, which renders neurons more vulnerable to ischemia [52], thereby indicating the roles of VCP/p97 in the regulation of mitochondrial function.

Under physiological conditions, VCP/p97, which is recruited by UBX1 to carbonyl cyanide 3-chlorophenylhydrazone (CCCP)-depolarized mitochondria, promotes mitochondrial autophagy for removal of damaged mitochondria and maintenance of mitochondrial fidelity [25]. VCP/p97 also mediates E3 ubiquitin ligase Parkin-induced Mfn1 degradation, and PINK1 degradation, to maintain mitochondrial proteostasis [53]. Under pathological conditions, the VCP/p97 mutant (*VCP/p97* R155C or *VCP/p97* R191Q) induces adenine nucleotide transferase dysregulation, resulting in a reduction in the rate of ADP or ATP translocation on the mitochondrial membrane [20]. Furthermore, VCP mutants migrate more slowly than endogenous VCP to the outer mitochondrial membrane, and also inhibit the localization of its cofactors Npl4 and p47 in damaged mitochondria, which collectively inhibits mitochondrial autophagy [54].

In the myocardial tissue, hypoxic-ischemic stimulation promotes the preferential accumulation of VCP/p97 in mitochondria [48]. Compared with wild-type mice, mitochondrial respiration capacity and ATP synthesis capacity in VCP/p97 transgenic mice are significantly enhanced, and the opening of mitochondrial permeability transition pore (mPTP) on mitochondria is consistently inhibited, thereby leading to considerable elimination of mitochondrial calcium overload caused by calcium influx [48]. A mechanistic study showed that VCP/p97 regulation of mitochondrial respiratory functions was dependent on iNOS. The effect of VCP/p97 overexpression on the enhancement of mitochondrial respiratory function is abolished with genetic deletion of iNOS in VCP/p97 transgenic mice, or by inhibition of iNOS activity with the iNOS inhibitor 1400W. The protective effects of VCP/p97 on mitochondrial calcium overload may partially rely on mitochondrial Ca^2+^ uptake protein 1 (MICU1) [55], which is an activator of mitochondrial Ca^2+^ unidirectional transporter (MCU), and is responsible for mitochondrial calcium uptake [56]. VCP/p97 physically associates with MICU1 through Parkin, and promotes post-translational protein degradation of MICU1, which may contribute to reduction in the frequency of mPTP opening and the damage caused by mitochondrial calcium overload (Figure 1).

Overexpression of VCP/p97 in transgenic mice reveals an important role in anti-ischemia/reperfusion injury by promoting iNOS production and by improving mitochondrial function. A VCP/p97 inhibitor (KUS121) was also shown to attenuate ischemia–reperfusion injury in various animal models, including murine and porcine ischemia and reperfusion injury models. Specifically, KUS121 treatment increases the level of ATP, reduces ER stress, and improves mitochondrial function, thereby reducing myocardial cell death caused by ischemia–reperfusion injury [57]. We hypothesize that this contradictory result may be associated with the method of activation and inhibition of VCP/p97 activity. First, VCP/p97 transgenic mice exhibit certain physiological processes other than VCP/p97 ATPase activity, including endoplasmic reticulum (ER)–associated degradation, lysosomal protein degradation, and so on [10], which may triggers systematic protective effects after ischemia/reperfusion injury, whereas KUS121 only inhibits VCP/p97ATPase activity. Second, VCP/p97 transgenic mice have undergone systemic changes since the embryonic stage compared with the short-term effects of KUS121, which may not provide sufficient time for adaptation. Therefore, both temporary inhibition of the ATPase activity of VCP/p97 after ischemia and generation of VCP/p97 transgenic mice before ischemia–reperfusion injury demonstrates strong anti-ischemia/reperfusion effects.

### VCP/p97 and hypertensive heart disease

**Figure 2.**
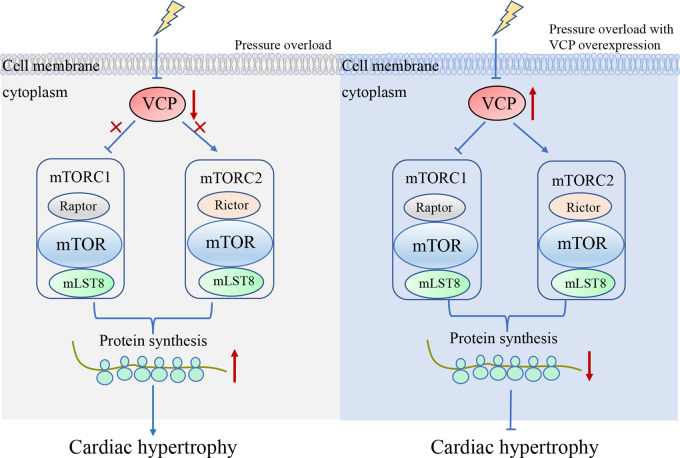
VCP's effects on pressure overload induced cardiac hypertrophy. Pressure overload inhibits VCP in cardiomyocytes, reduces VCP's activation of mTORC2 signaling pathway and its inhibitory effect on mTORC1 signaling pathway, and ultimately leads to increased protein synthesis and cell apoptosis; However, overexpression of VCP can effectively restored the activation of mTORC2 signaling pathway and inhibited mTORC1 signaling pathway, and resisting myocardial hypertrophy caused by pressure overload.

Hypertension usually leads to cardiac hypertrophy, a disease manifested by hypertrophy of single cardiomyocytes and enhanced rates of protein synthesis [58]. Myocardial hypertrophy is an independent pathological risk factor for many cardiovascular diseases, including myocardial infarction [59] and heart failure [60]. mTOR (mammalian target of rapamycin) is an evolutionarily conserved serine/tyrosine kinase which interacts with specific adaptor proteins to form two different macromolecular complexes, called mTORC1 (mTOR complex 1) and mTORC2 (mTOR complex 2) [61]. These two complexes are essential for the development of adaptive myocardial hypertrophy caused by pressure overload. The absence of mTORC2 damages compensatory myocardial hypertrophy [62], and partial inhibition of mTORC1 may eliminate pathological cardiac hypertrophy and improve left ventricular function [63]. Zhou N et al. [64] found that VCP/p97 expression is time- and dose-dependently reduced in pressure overload. And overexpression VCP/p97 protects heart from cardiac hypertrophy, supported by the facts that VCP/p97 transgenic mice shown relatively normal cardiac structure and cardiac function compared with wild-type mice in the presence of cardiac pressure overload [64] (Figure 2). Pressure overload suppresses the expression of VCP/p97 which activated mTORC2 and attenuates the inhibitive effect of VCP/p97 on mTORC1 signaling, subsequently promotes the protein synthesis pathway (Figure 2). While VCP/p97 overexpression restores the pressure overload-suppressed VCP/p97 and represses the pressure overload-induced activation of mTORC1, protects the heart against pressure overload-induced cardiac hypertrophy [65] (Figure 2).

In the progression of cardiac hypertrophy, there are multiple molecular alterations [66]. One of these alterations involves changes to Akt activity, which plays a crucial role in the regulation of a variety of cellular processes ranging from cell survival to aging [67]. As described previously, Akt activates VCP/p97 in persistent hypoxic states [68]. Interestingly, in a study by Zhou et al.[64], VCP/p97 also promotes Akt phosphorylation under physiological conditions. Two-dimensional gel electrophoresis and mass spectrometry combined with immunoprecipitation experiments have confirmed that VCP/p97 and Akt bind to each other [69]. As a kinase protein, Akt can directly phosphorylate VCP/p97, whereas VCP/p97 is not a kinase protein, and does not possess phosphorylation ability. Given the fact that the activity of mTORC2 is up-regulated in VCP/p97 transgenic mice, and that Akt acts downstream of mTORC2, it is suggested that VCP/p97 may first activate mTORC2, and then promote Akt phosphorylation [64]. This finding indicates that interactions between Akt and VCP/p97 may involve both direct and indirect regulation, and these two proteins may exhibit different modes of action under different physiological conditions. Since Akt is a central protein involved in many cellular processes in cardiac hypertrophy, further studies focusing on the relationship between VCP/p97 and Akt will help to further reveal the roles of VCP/p97 in cardiac hypertrophy.

However, the protective effects of VCP/p97 in myocardial hypertrophy remain controversial. A recent study showed that overexpression of VCP/p97 did not alleviate the progression of myocardial hypertrophy caused by pressure overload. After 4 to 8 weeks of transverse aortic constriction (TAC) surgery, the left ventricular internal diameter and fractional shortening of VCP/p97 transgenic mice were not significantly different from those of control mice [10]. The authors assumed that VCP/p97 represented 1% of the total protein; hence, the effect of overexpressing VCP/p97 on the myocardium was minimal. It is worth noting that in this study, mice in the control group did not exhibit significant differences in the cardiac structure and function after receiving TAC surgery for 4 and 8 weeks, which prompted us to question the reliability of the cardiac pressure overload model. In general, long-term pressure overload inevitably leads to dramatic changes in the cardiac structure and function. Therefore, conclusions based on this model are also debatable. However, whether VCP/p97 exerts protective effects in myocardial hypertrophy requires further investigation.

## Summary and outlook

Cardiovascular diseases, including myocardial infarction and heart failure, are important causes of high mortality rates in the elderly [70]. Traditional studies have attempted to identify risk factors related to the occurrence of cardiovascular diseases, and to reduce such risks by means of therapeutic drugs or gene knockout [71]. In recent years, it has been proven that endogenous protective factors within cardiomyocytes considerably help to reduce the risk of cardiovascular disease. VCP/p97 is a typical endogenous protective factor, which has long been considered to be associated with physiological processes that maintain protein homeostasis [72]. Recent studies have shown that VCP/p97 may be a potential therapeutic target in cardiovascular diseases. Reduction or abnormal function can cause a variety of diseases. The activation or overexpression of VCP/p97 can effectively delay the progression of diseases. In particular, overexpression of VCP/p97 can effectively increase the level of high-energy phosphate in the myocardium, resist mitochondrial calcium overload, and promote cell survival [48].

Existing research suggests that VCP/p97 has a strong potential to be a new therapeutic target for ischemic-reperfusion injury and pressure overload-induced cardiac hypertrophy, although there remain some opposing views. If VCP/p97 is to be seriously considered, then more research should be conducted to determine whether VCP/p97 has general myocardial protective effects, and the mechanisms by which it is involved in such processes. In particular, the role of VCP/p97 in protection against non-ischemic heart disease, including diabetic cardiomyopathy and idiopathic dilated cardiomyopathy should be elucidated. Additionally, many cardiovascular diseases caused by pressure overload, including pathological cardiac hypertrophy, result in protein homeostasis imbalance [73]. The mechanism by which VCP/p97 maintains myocardial protein homeostasis should be studied. Moreover, since the functions of mitochondria are diverse, it is possible that VCP/p97 may also regulate other aspects of mitochondria (including redox balance, glucose oxidation, and lipid oxidation), in spite of respiratory function and calcium hemostasis. Further research is needed to explore the complex relationships involving mitochondria and VCP/p97.

*VCP/p97* mutations affect cardiac function. As mentioned before, mice that overexpress mutant VCP/p97 (*VCP/p97* K524A) are prone to cardiomyopathy under cardiac pressure overload [10] due to loss of ATPase activity. The ATPase activity of VCP/p97 is a basic requirement for its normal function because it provides the energy required for extraction of ubiquitinated proteins from membrane structures (such as the endoplasmic reticulum) [74]. In line with this canonical view, the mutation of VCP/p97 involving Lys524 undermines the efficiency of protein clearance in the ERAD pathway, and affects nuclear integrity. However, this view has been challenged recently. A new substance (KUS121), which inhibits the ATPase activity of VCP/p97, has been shown to exert protective effects in *in vivo* myocardial infarction models, and in *in vitro* cardiomyocyte hypoxia models [57], which is contradictory to a study involving *VCP/p97* K524A transgenic mice [10]. Although the structure of VCP/p97 has been clearly determined, more research is needed to clarify discrepancies in particular pathophysiological processes.

In summary, VCP/p97 plays important roles in the cardiovascular system. It may become a promising target for the treatment of cardiovascular diseases; however, the underlying molecular mechanisms await further investigation.

## Perspectives

- *Importance of the field:* VCP/p97 is a type II AAA+ ATPase that is conserved and abundantly expressed in a variety of tissues and cells, and is involved in a variety of physiological processes. This protein is increasingly associated with human diseases including cardiovascular system diseases, which has inspired people to further explore its important role in cardiovascular system.
- *Summary of current thinking:* VCP/p97 deficiency damages myocardial fibers and induce heart failure, while VCP/p97 overexpression improves ischemia–reperfusion injury and pressure overload induced cardiac hypertrophy. Emerging evidence also indicates VCP/p97 plays an important role in maintaining normal mitochondrial respiratory function of cardiomyocytes and promoting its survival.
- *Future directions:* Many unresolved problems remain about the role of VCP/p97 in the cardiovascular field. For example, the protective effect of VCP/p97 on myocardial hypertrophy caused by ischemia–reperfusion injury and pressure overload is still controversial, studies are particularly required to understand its underlying molecular mechanisms.

## Abbreviations

ALS: amyotrophic lateral sclerosis
CCCP: carbonyl cyanide 3-chlorophenylhydrazone
Cdc48: cell division cycle 48
ER: endoplasmic reticulum
ERAD: endoplasmic reticulum-associated degradation pathway
IBMPFD: inclusion body myopathy, Paget disease, neurological components frontotemporal dementia
iNOS: inducible NO synthase
MCU: mitochondrial Ca^2+^ unidirectional transporter
MICU1: mitochondrial Ca^2+^ uptake protein 1
mPTP: mitochondrial permeability transition pore
mTOR: mammalian target of rapamycin
mTORC1: mTOR complex 1
mTORC2: mTOR complex 2
Npl4: nuclear protein localization protein 4
TAC: transverse aortic constriction
TER: ATPase transitional endoplasmic reticulum ATPase
UBX: ubiquitin regulatory X
UBXD1: UBX domain-containing protein 1
VBM: VCP/p97-binding motif
VCP/p97: valosin-containing protein
VIM: VCP/p97-interacting motif
